# Supplementary material for: Magnetic field filtering of the boundary supercurrent in unconventional metal NiTe2-based Josephson junctions
Source: Nat Commun. 2024 Mar 30;15:2785. doi: 10.1038/s41467-024-47103-z (PMC10981750; doi:10.1038/s41467-024-47103-z)
Supplement: Supplementary file 1 — Supplementary Information [file 41467_2024_47103_MOESM1_ESM.pdf]

Supplementary Information for

Magnetic field filtering of the boundary supercurrent in unconventional metal $NiTe_2$-based Josephson junctions

Tian Le[1#], Ruihan Zhang[1,2#], Changcun Li[3], Ruiyang Jiang[1,2], Haohao Sheng[1,2], Linfeng Tu[1,4], Xuewei Cao[4], Zhaozheng Lyu[1,5], Jie Shen[1,6], Guangtong Liu[1,5,6], Fucai Liu[3,7*], Zhijun Wang[1,2*], Li Lu[1,2,5,6*], Fanming Qu[1,2,5,6*]

[1] Beijing National Laboratory for Condensed Matter Physics, Institute of Physics, Chinese Academy of Sciences, Beijing 100190, China

[2] School of Physical Sciences, University of Chinese Academy of Sciences, Beijing 100049, China

[3] School of Optoelectronic Science and Engineering, University of Electronic Science and Technology of China, Chengdu 611731, China

[4] School of Physics, Nankai University, Tianjin 300071, China

[5] Hefei National Laboratory, Hefei 230088, China

[6] Songshan Lake Materials Laboratory, Dongguan, Guangdong 523808, China

[7] Yangtze Delta Region Institute (Huzhou), University of Electronic Science and Technology of China, Huzhou 313009, China

[#] These authors contributed equally to this work.

[*] Emails: fucailiu@uestc.edu.cn; wzj@iphy.ac.cn; lilu@iphy.ac.cn; fanmingqu@iphy.ac.cn

Content

Section I. Extraction of the supercurrent density profile.

Section II. Misalignment between the sample and in-plane magnetic field.

Section III. In-plane magnetic field suppression of the bulk supercurrent.

Section IV. Analysis of the critical side-surface supercurrent (side-surface-I_c) under B_y.

Section V. SIP for D1.

Section VI. Theoretical calculation.

Section VII. The localization length of the hinge states.

Section VIII. Another junction that does not cover the hinges/side surfaces.

Section IX. Train of thought on the existence of hinge states.

Section I. Extraction of the supercurrent density profile.

In this section, we will introduce the Dynes-Fulton approach for converting the superconducting interference pattern (SIP) $I_c(B_z)$ to the supercurrent density profile $J_s(y)$, taking Figs. 2a and 2b in the main text as an example.

In our configurations, the supercurrent density varies along the y direction. The separation of the two electrodes is L and the width of each electrode is t. Considering the magnetic flux focusing effect of the superconducting electrodes, the effective length of the junction is $L_{eff} = L+t$. The critical supercurrent $I_c(B_z)$ is extracted following the whitish envelope in Fig. 2a which was replotted as Fig. S1a. The red curve in Fig. S1a illustrates $I_c(B_z)$. The experimentally observed $I_c(B_z)$ is the magnitude of the integration of $J_s(y)$. Assuming the normalized magnetic field unit of $\kappa = 2\pi L_{eff} B_z/\Phi_0$, where $\Phi_0 = h/2e$ is the flux quantum (h is the Planck constant and e is the elementary charge), $I_c(B_z)$ can be replaced by the complex critical supercurrent function $\mathfrak{I}_c(\kappa)$:

$$I_c(\kappa) = |\mathfrak{I}_c(\kappa)| = \left|\int_{-\infty}^{\infty} J_s(x) e^{i\kappa y} dy\right|.$$

Considering an even supercurrent density $J_{even}(y)$ with a symmetric distribution, the

odd part of $e^{i\kappa y}$ vanishes from the integral, and we can obtain

$$\mathfrak{I}_c(\kappa) = I_{even}(\kappa) = \int_{-\infty}^{\infty} J_{even}(y)\cos(\kappa y)dy.$$

$\mathfrak{I}_c(\kappa)$ in the above formula alternates between positive and negative values at each zero-crossing. Because $I_{even}(\kappa)$ usually dominates in the observed critical supercurrent except at its minima, it can be roughly obtained by multiplying $I_c(\kappa)$ with a flipping function as shown in Fig. S1b, where the sign is switched between adjacent lobes of the whitish envelope. When $I_{even}(\kappa)$ is minimal, $I_c(\kappa)$ is dominated by the odd component: $I_{odd}(\kappa) = \int_{-\infty}^{+\infty} J_{odd}(y)\sin(\kappa y)dy$. $I_{odd}(\kappa)$ can then be approximated by interpolating between the minima of $I_c(\kappa)$, and flipping sign between lobes as shown in Fig. S1c. A Fourier transform of the complex critical supercurrent function $\mathfrak{I}_c(\kappa) = I_{even}(\kappa) + iI_{odd}(\kappa)$ is:

$$J_S(y) = \left|\frac{1}{2\pi}\int_{-W/2}^{W/2} \mathfrak{I}_c(\kappa)e^{-i\kappa y}d\kappa\right|,$$

which produces the supercurrent density profile as shown in Fig. S1d (the same as Fig. 2b).

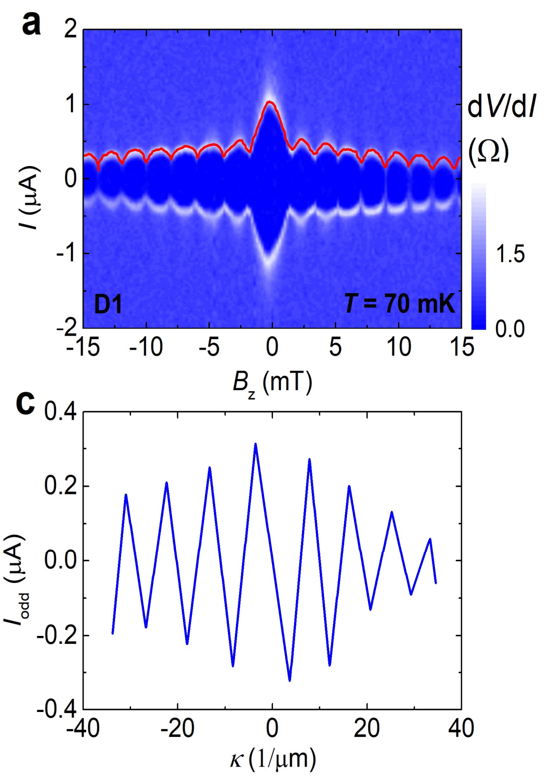

Fig. S1. **a**, SIP for D1, the same as Fig. 2a in the main text. The red curve illustrates $I_c(B_z)$ taken following the whitish envelope for an example. **b**, The even part of $I_c(\kappa)$ recovered from the red curve in **a**. **c**, The odd part of $I_c(\kappa)$ recovered from the red curve in **a**. **d**, Supercurrent density profile for $I_c(B_z)$ in **a**.

Section II. Misalignment between the sample and in-plane magnetic field.

We note that the misalignment between the sample and in-plane magnetic field could induce the edge dominated current profile. However, this is not the case in our work and the misalignment has been corrected when an in-plane magnetic field is applied. We can estimate the misalignment angle from the superconducting interference pattern (SIP) using the central peak at finite $B_{x,\,y}$. When an in-plane magnetic field is applied, the x-axis of the data shown in the main text has been corrected to eliminate the B_z component induced by $B_{x,\,y}$. For example, Fig. S2a shows the original data on D1 at B_x = 0.1 T, which indicates a shift of the central peak of around 0.5 mT, i.e., the B_z component is ~ 0.5 mT. Therefore, the misalignment angle should be close to 0.3 degree, and the B_z component at B_x = 0.2 T is ~ 1 mT, which can be corrected. The x-axis of the 2D color plots shown in Figs. 2c, 2d, 3b, 3d, 4b-e, and 5b has been corrected using this

approach, and some more examples are shown in Fig. S2.

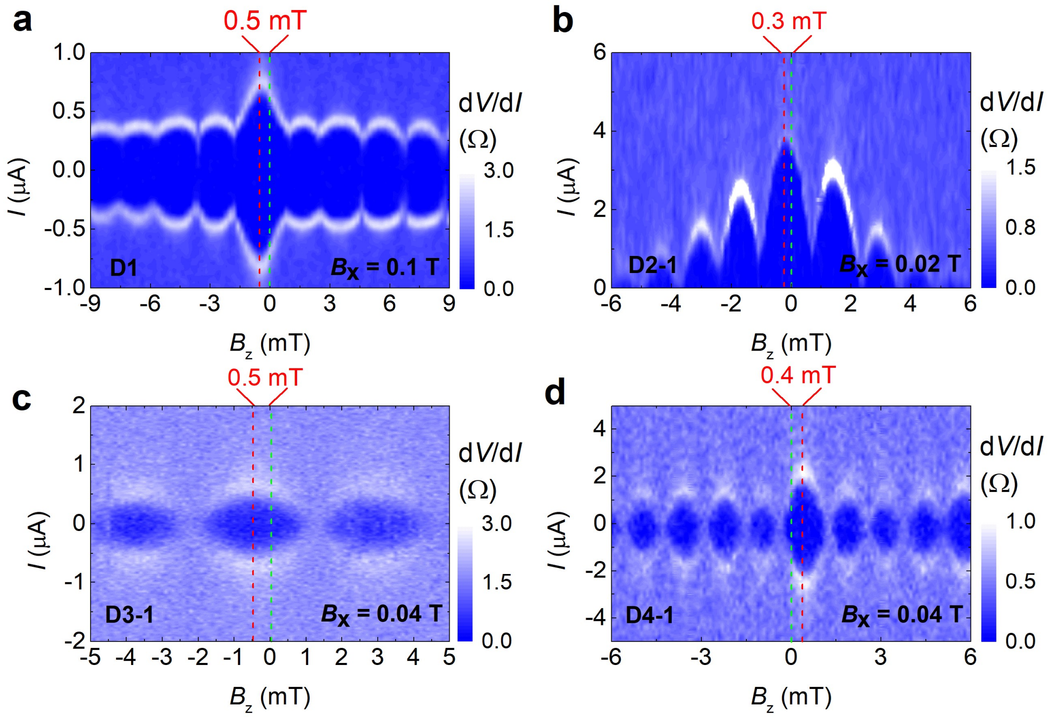

Fig. S2. The original SIPs for different devices at finite B_x without eliminating the B_z component. The two dashed lines indicate the B_z component due to the misalignment.

Section III. In-plane magnetic field suppression of the bulk supercurrent.

We note that the B_x for killing the bulk supercurrent on D1 is much larger than D2, D3 and D4. We think it is caused by the detailed fabrication process (e.g., the quality of the superconductor films), because D2, D3 and D4 are fabricated simultaneously. However, D1 was fabricated and measured around six months earlier than them. In Fig. S3, we present another device D5, which was fabricated together with D1, and it also shows a larger B_x for killing the bulk supercurrent.

On the other hand, why the supercurrent on bulk states disappears at such low in-plane field? And why the suppression rate differs for B_x and B_y? We next discuss the possible mechanisms.

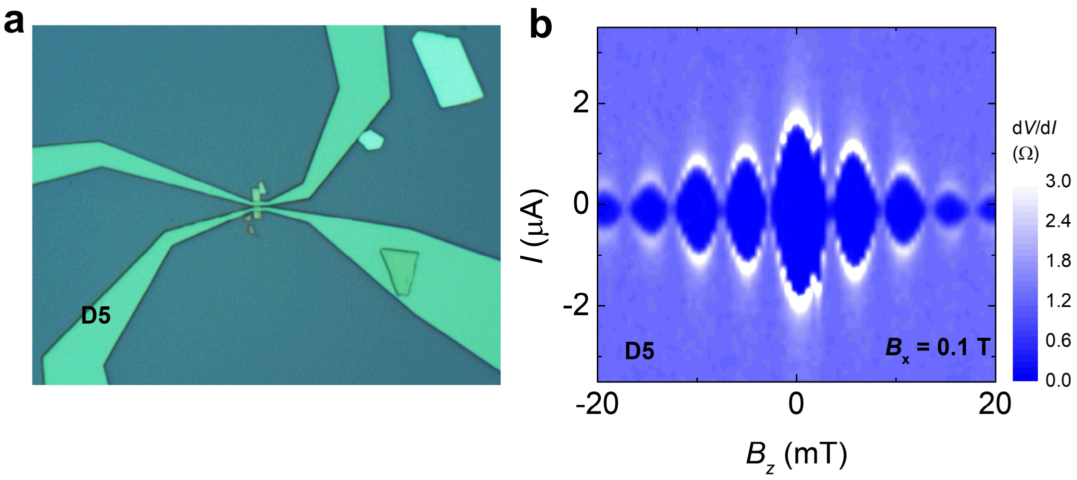

Fig. S3. **a**, The optical image for D5. **b**, SIP for D5 under $B_x = 0.1$ T.

1. It could be contributed to the Gaussian-like decay of the bulk supercurrent, given by $I_c(B_x) = I_c(0)e^{-0.145\Gamma_{sf}/\epsilon_T} \approx I_c(0)e^{-B^2/2\sigma^2}$[1]. Here Γ_{sf} is the spin-flip scattering, ϵ_T is the Thouless energy and σ is the effective decay coefficient. When the spin-flip length $L_{sf} = \sqrt{\hbar D/2\Gamma_{sf}}$ (D is the diffusion coefficient in the normal metal) becomes smaller than the length L of the SNS junction (L could be the separation of the electrodes), spin-flip scattering could act as a pair-breaking mechanism for the Cooper pairs, dominating the decay of supercurrent. In such case, Γ_{sf} is proportional to the square of magnetic field B^2. Based on this scenario, it is possible for the supercurrent on bulk states to vanish at low in-plane fields. We noticed that the critical in-plane field $H_{c2}^{||}$ of the bulk states in Cd_3As_2-based planar Josephson junctions is also small, which is around 0.1 T in Ref. 2[2]. However, the data shows an anisotropic suppression on the bulk supercurrent. As seen in Fig. 4b, $B_x = 0.04$ T gives rise to $I_c = 1.6$ uA. However, $B_y = 0.04$ T gives rise to $I_c = 7$ uA. We noticed that the B^2 dependence not only relies on the thickness W, but is also associated with the diffusion coefficient D. Therefore, while W is the same for B_x and B_y, the anisotropic suppression of the bulk supercurrent requires D to be anisotropic in $NiTe_2$, if this mechanism indeed applies. As of now, no reports on the anisotropic diffusion coefficient in $NiTe_2$ have been published, indicating that the "orbital spin-flip" mechanism may not capture the anisotropic suppression.

2. As reported in Ref. 3[3], the application of an in-plane magnetic field can lead to Zeeman splitting, resulting in an exponentially suppressed critical current, aligning with the observed fast bulk suppression rate. Moreover, this Zeeman-driven mechanism is also intricately linked to the diffusion coefficient (D). But this "Zeeman-driven FFLO" mechanism should be symmetric towards B_x and B_y. If it applies to the anisotropic suppression rates between B_x and B_y in our work, again, anisotropic D in $NiTe_2$ is required, which is unknow yet.

3. Another mechanism is related to spin-orbital suppression. In Ref. 4[4], finite-momentum Cooper pairing was observed in HgTe quantum wells in an in-plane magnetic field. Spin-orbit coupling (SOC) resulted from structural inversion asymmetry or bulk inversion asymmetry is required, and the performance of the Josephson junction depends on the type of SOC. Detailed calculations involving SOC and the geometry of the junction did show a fast and anisotropic suppression of the bulk supercurrent in an in-plane magnetic field. The spin-orbital suppression is a manifestation of the interplay between in-plane magnetic field and SOC, and the anisotropy mainly comes from the difference between B_x and B_y on the flux penetration. For the scenario when B_x aligns parallel to the current direction, it allows the magnetic flux to penetrate the superconducting electrodes and subsequently suppresses the supercurrent. This mechanism could indeed effectively account for both our observed anisotropic in-plane suppression behavior and the remarkably low critical in-plane field along B_x, while the type of SOC requires further investigation. This is mostly likely the physical mechanism. In addition, we would like to note that the observed boundary supercurrent in both zero and finite in-plane magnetic fields is particular in our work.

4. It could be a common phenomenon that has been reported on other Josephson junctions with a similar electrode configuration[5]. We might attribute it to the vimineous shape of the electrodes that exhibit anisotropic demagnetization N[6]. It is natural to assume the proximity-induced superconducting region beneath the electrodes to be a stripe shape. The effective demagnetization can be approximated as[7]:

$$N^{-1} = 1 + \frac{3}{4}\frac{l_{\parallel}}{l_{\perp}}$$

where the symbol $l_{\parallel}$ represents the length along the magnetic field direction, $l_{\perp}$ is the length perpendicular to the magnetic field. Suppose $l_{\parallel}$ and $l_{\perp}$ are comparable to the size of the electrodes on the sample (2.1 μm × 0.5 μm), and N is estimated to be around 0.84 for B_x and 0.24 for B_y, which produce a large anisotropy.

Section IV. Analysis of the critical side-surface supercurrent (side-surface-I_c) under B_y.

If the boundary supercurrent originates from the side surfaces (rather than the hinges), we can calculate its value at a certain B_y through the Fraunhofer-like decay curve $I_c(B_y)/I_c(B_y = 0\text{ T}) = |\sin(\pi\Phi/\Phi_0)/(\pi\Phi/\Phi_0)|$. Note that this is under the ideal assumption of uniform supercurrent distribution along the rectangular side surface. To calculate the critical supercurrent, we need to supress the contribution of the bulk supercurrent, which can be achieved by applying a small B_z to render the Fraunhofer-like $1/|B|$ decay of the bulk supercurrent itself. Therefore, we inspect the side lobes of the SIP in Fig. 2a in the main text, where the bulk supercurrent has been supressed due to the Fraunhofer-like decay in B_z. We assign the height of the side lobes (excluding the central lobe) of the SIP in Fig. 2a as I_c of the side-surface, i.e., side-surface-$I_c(B_y = 0$ T). Using the equation $I_c(B_y)/I_c(B_y = 0\text{ T}) = |\sin(\pi\Phi/\Phi_0)/(\pi\Phi/\Phi_0)|$, we can calculate the side-surface-I_c at $B_y = 0.2$ T. In the main text, we assume that the effective junction length of the side surface is comparable to the separation of the electrodes, i.e., ~300 nm. In fact, it could be underestimated if considering the flux focusing effect of the electrodes. We thus test several conditions here, as shown in Fig. S5, and side-surface-I_c at $B_y = 0.2$ T is always smaller than the height of the first side lobe, which is ~$0.21I_c(B_y = 0$ T). However, the experimental boundary-$I_c(B_y = 0.2$ T) is much larger than $0.21I_c(B_y = 0$ T) as shown in Fig. S4

The assumption of uniform supercurrent along the side surfaces (if exist), which follows

the ideal Fraunhofer pattern and the $1/|B|$ decay, may not hold for various kinds of reasons. The mechanisms include, e.g., the self-field effect when the critical current is very large[8], the irregular shape of sandwich-like junctions[9], and an asymmetry in the current-phase relation[5]. These examples may not be directly applicable to our rectangular side-surface-based junctions, though, they do demonstrate different scenarios of the non-Fraunhofer patterns. Of course, there are also planar Josephson junctions with a regular rectangular shape that exhibit an approximate $1/|B|$ decay, such as Ref. 4[4], Ref. 10[10], Ref. 11[11], etc. Furthermore, nonuniform supercurrent distribution stemming from factors like imperfect fabrication process, long Josephson junctions, and different current channels can cause deviations in the magnitude of the lobes from the ideal $1/|B|$ decay. Indeed, we cannot exclude the potential for non-ideal Fraunhofer patterns in our devices. Nevertheless, a faster decay than the dada in Fig. S4 is likely expected if side surfaces indeed exist.

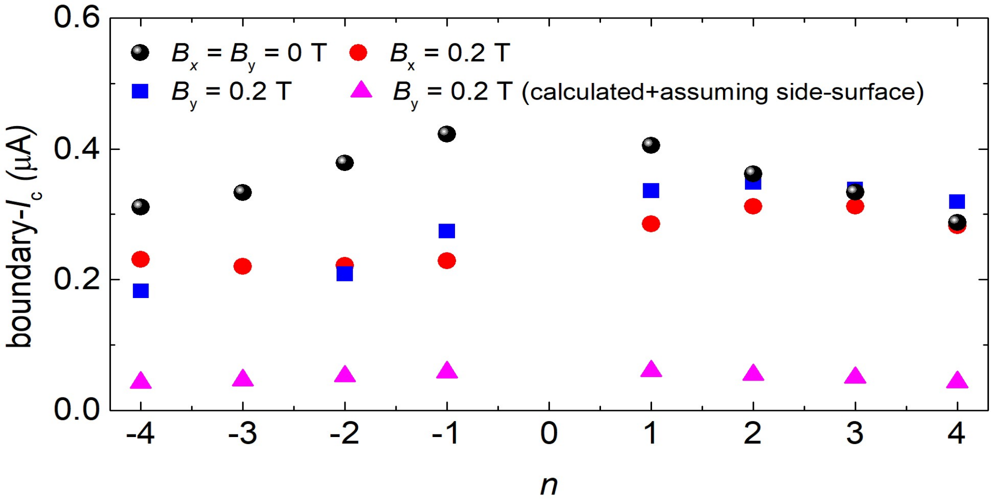

Fig. S4. The black balls denote the extracted boundary-I_c from each center of the side lobes in Fig. 2a with $B_x = B_y = 0$ T. The red cycles and blue squares denote the extracted boundary-I_c from each center of the side lobes in Fig. 2d and Fig. S6 with $B_x = 0.2$ T and $B_y = 0.2$ T, respectively. The pink triangles represent the calculated boundary-I_c for each center of side lobes when $B_y = 0.2$ T, if assuming the existence of side-surface supercurrent that follows the ideal Fraunhofer pattern. n denotes the serial number of the side lobes.

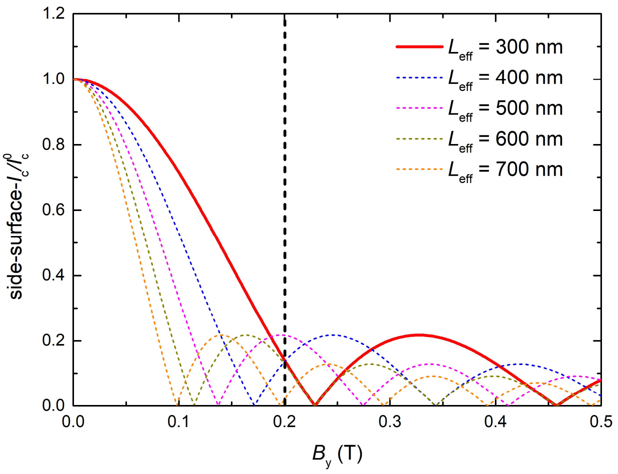

Fig. S5. Simulation of Fraunhofer-like curves for different effective junction lengths assuming an ideal side-surface supercurrent.

Section V. SIP for D1.

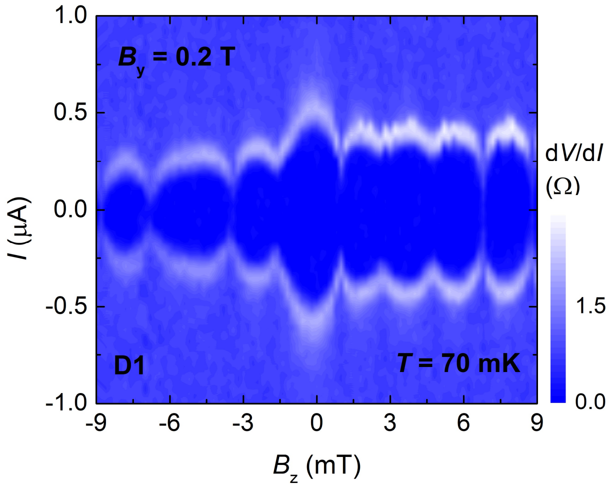

Fig. S6. SIP for D1 at $B_y = 0.2$ T.

Figure S6 shows the SIP for D1 at $B_y = 0.2$ T, which was used to extract the boundary-I_c at $B_y = 0.2$ T for the side lobes presented in Fig. S4.

Section VI. Theoretical calculation.

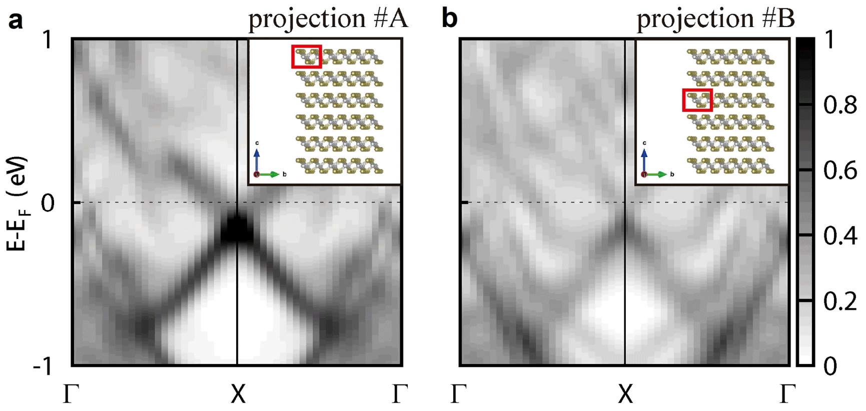

Fig. S7. The bands of **a**, hinge state and **b**, side-surface state. Compared with the side-surface atoms' projection (projection #B), the hinge atoms' projection (projection #A) contributes more around E_F, which indicates that the band contribution near the Fermi level is dominated by the hinge state rather than side-surface state.

Section VII. The localization length of the hinge states.

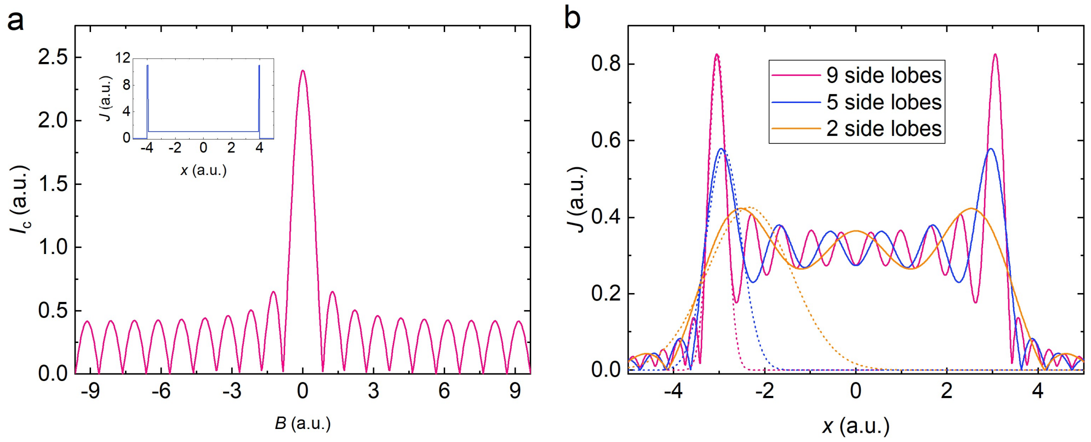

Fig. S8. **a**, Simulation of the SIP (with 9 side lobes shown) for the current density distribution shown in the inset. **b**, Current density profiles extracted from a, assuming 9, 5 and 2 side lobes, respectively. The dashed lines are fittings using the Gaussian function.

In Josephson junctions, in principle, the localization length of the edge/hinge states could be extracted from the current density profiles obtained by the Dynes-Fulton approach. As many references have done, the length is 100 nm for the hinge states in WTe_2[12], 400 – 600 nm for the hinge states in Cr_3As_2[13], 300 – 400 nm for the edge states in HgTe/CdTe[11], and 220 nm for the edge states in graphene[14]. Using the same method, we could estimate the localization length, which is around 150 nm for Fig. 2b, 190 nm for Fig. 3b, and 270 nm for Fig. 3d. These values are similar to that in the above references.

However, we should note that the resolution of this method is intimately related to the number of measured side lobes in the SIP, and thus the extracted length is not an accurate value, usually largely overestimated. One typical example can be seen from Figs. 3b and 3d, which present a large difference in the width of the current-density peaks due to the different number of side lobes. In order to describe the limitation of the Dynes-Fulton approach in extracting the localization length of the hinge/edge states, we show the simulation results in Fig. S8. We assume that the localization length of the hinge states is $d = 1\%W$ (W is the width of the junction), as shown by the inset of Fig.

S8a. The theoretical SIP containing SQUID-like component is shown in Fig. S8a. Then we take part of this SIP to extract the current density profile, as shown in Fig. S8b for 2, 5, and 9 side lobes on each side of the central lobe, respectively. The dashed lines are the Gaussian fitting of the peaks, by which the width can be obtained. However, the width is $5d$ for 9 side lobes, $9.5d$ for 5 side lobes, and $23d$ for 2 side lobes. Therefore, this value extracted from the Dynes-Fulton approach is an upper bound due to the limited number of side lobes in the SIP, and this approach is qualitative instead of quantitative in realistic experiments.

In fact, the localization length of hinge states should be small, on the scale of nanometers. For instance, the higher-order topological hinge states in Bi have been studied by STM and transport[15-17]. For the transport measurement, the hinge states can be recognized due to the different enclosed areas in a SQUID device for a narrow Bi nanowire with a thickness and width between 30 and 200 nm. So, the localization length should be much less than 30 nm. A more direct and accurate measurement is the STM, which presented the nanometer-scale hinge states clearly. Therefore, the extracted 100 nm-wide hinge states in WTe_2[12], and the > 100 nm-wide hinge states in our work, should both be largely overestimated and both be in the nanometer scale.

Section VIII. Another junction that does not cover the hinges/side surfaces.

We note that the exfoliated $NiTe_2$ nanoplates usually have parallel long edges, indicating a certain crystal direction. The junctions (D1, D2-1, D3-1, D4-1, and D5) that cover the sample hinges/side surfaces and present boundary supercurrent are

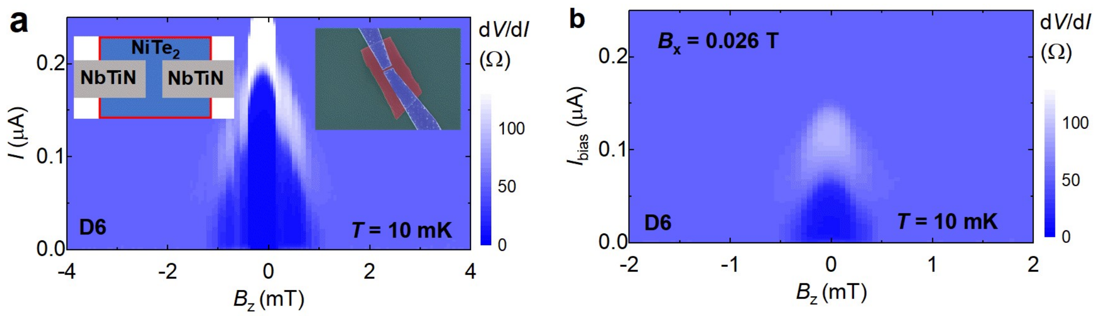

Fig. S9. **a**, SIP for D6 at 10 mK without in-plane magnetic field. **b**, SIP for D6 at 10 mK under $B_x = 0.026$ T.

vertical to the parallel long edges of the sample, and the junction (D2-2) that does not cover the hinges/side surfaces and does not show boundary supercurrent is parallel to the parallel long edges. In order to address the issue of possible anisotropy, we fabricated a junction (D6) that is vertical to the parallel long edges of the nanoplate, as shown in the inset of Fig. S9. We observed similar patterns to junction D2-2 (Fig. 5), showing neither boundary supercurrent nor anisotropic behavior. Please note that the current density is not uniform as shown by the right inset of Fig. 5a, and thus the SIP is distorted from the standard Fraunhofer pattern and side lobes can be hardly observed, as discussed in the main text.

Section IX. Train of thought on the existence of hinge states.

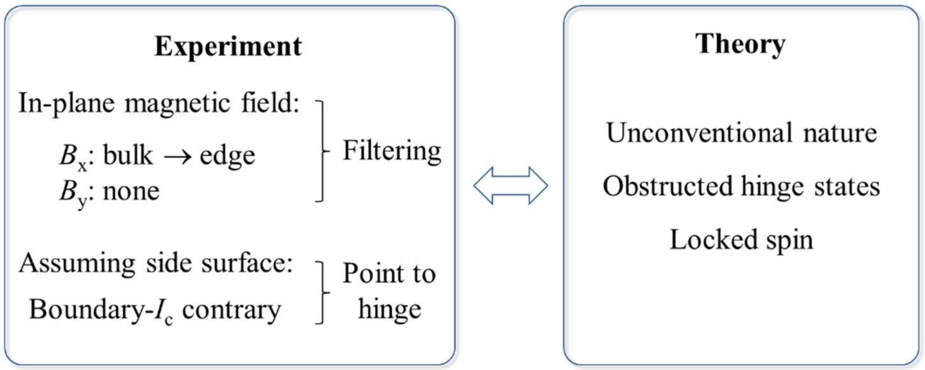

Fig. S10. Train of thought on the existence of hinge states.

How we achieved the explanation of the existence of hinge states is incorporating our experimental observations with the theoretical calculations. To show an overview of our train of thought, we summarized the results roughly in Fig. S10.

Experimentally, we observed the transition from the bulk to the edge when an in-plane magnetic field (B_x) parallel to the current is applied, which we call the filtering effect. However, in-plane magnetic field (B_y) perpendicular to the current does not show such effect. To find the clue on the origin of the edge state, i.e., the side surface or the hinge, we assume that it is the side surface and compare with the experimental data. But we find contrary between the experimental boundary-I_c at $B_y = 0.2$ T and the side-surface scenario, as shown in Fig. S4. Therefore, it indicates the hinge supercurrent. Please note

that the assumption of ideal Fraunhofer pattern may not hold due to various mechanisms, as discussed earlier.

Theoretically, our detailed calculations show that $NiTe_2$ has the unconventional nature of charge mismatch, which gives rise to the obstructed hinge states. Importantly, due to the existence of time reversal symmetry and mirror symmetry (m_x), the electron spins of the hinge states are locked to be in the plane perpendicular to the hinges (along the x direction), which could explain the filter effect of the supercurrent under B_x. As illustrated in Fig. 6h, this locking protects the Cooper pairs of the hinge states from undergoing depairing in B_x. In contrary, the spins of the bulk states are randomized without such protection, and hence the filtering of hinge supercurrent can be observed. B_y represents the magnetic field perpendicular to the current. In this case, the acquired Zeeman energy to break Cooper pairs for the spins of the hinges is almost the same as that for the bulk. As a result, the Cooper pairs are not protected by the spin-momentum locking. Therefore, the magnetic field filtering of supercurrent is absent for B_y.

In summary, combining the experimental and theoretical results, we reached a self-consistent conclusion that the supercurrent is carried by hinge states. On the other hand, the side-surface scenario contradicts with both the experimental data and the theoretical calculations, and we do not know how to explain the filtering effect merely based on the edge accumulation or other effects, like in graphene or Bi_2O_2Se systems[14,18,19].

References

1 Hammer, J., Cuevas, J. C., Bergeret, F. & Belzig, W. Density of states and supercurrent in diffusive SNS junctions: Roles of nonideal interfaces and spin-flip scattering. *Phys. Rev. B* **76**, 064514, (2007).

2 Li, C.-Z. *et al.* Fermi-arc supercurrent oscillations in Dirac semimetal Josephson junctions. *Nat. Commun.* **11**, 1-7, (2020).

3 Dvir, T. *et al.* Planar graphene-$NbSe_2$ Josephson junctions in a parallel magnetic field. *Phys. Rev. B* **103**, 115401, (2021).

4 Hart, S. *et al.* Controlled finite momentum pairing and spatially varying order parameter in

proximitized HgTe quantum wells. *Nat. Phys.* **13**, 87-93, (2017).

5 Baumgartner, C. *et al.* Supercurrent rectification and magnetochiral effects in symmetric Josephson junctions. *Nat. Nano.* **17**, 39-44, (2022).

6 Mi, X. *et al.* Relevance of sample geometry on the in-plane anisotropy of $Sr_xBi_2Se_3$ superconductor. *arXiv: 2110.14447*, (2021).

7 Prozorov, R. & Kogan, V. G. Effective demagnetizing factors of diamagnetic samples of various shapes. *Phys. Rev. Appl.* **10**, 014030, (2018).

8 Kim, M. *et al.* Strong proximity Josephson coupling in vertically stacked $NbSe_2$–graphene–$NbSe_2$ van der Waals junctions. *Nano Lett.* **17**, 6125-6130, (2017).

9 Chen, X., Poortvliet, M., van der Molen, S. J. & de Dood, M. J. Interface shape dependent interference patterns of $NbSe_2$ heterostructure Josephson junctions. *Phys. Rev. B* **107**, 094522, (2023).

10 Qu, F. *et al.* Strong superconducting proximity effect in Pb-Bi_2Te_3 hybrid structures. *Sci. Rep.* **2**, 1-5 (2012).

11 Hart, S. *et al.* Induced superconductivity in the quantum spin Hall edge. *Nat. Phys.* **10**, 638-643, (2014).

12 Choi, Y.-B. *et al.* Evidence of higher-order topology in multilayer WTe_2 from Josephson coupling through anisotropic hinge states. *Nat. Mater.* **19**, 974-979, (2020).

13 Li, C.-Z. *et al.* Reducing electronic transport dimension to topological hinge states by increasing geometry size of Dirac semimetal Josephson junctions. *Phys. Rev. Lett.* **124**, 156601, (2020).

14 Allen, M. T. *et al.* Spatially resolved edge currents and guided-wave electronic states in graphene. *Nat. Phys.* **12**, 128-133, (2016).

15 Schindler, F. *et al.* Higher-order topology in bismuth. *Nat. Phys.* **14**, 918-924, (2018).

16 Murani, A. *et al.* Ballistic edge states in Bismuth nanowires revealed by SQUID interferometry. *Nat. Commun.* **8**, 15941, (2017).

17 Jack, B. *et al.* Observation of a Majorana zero mode in a topologically protected edge channel. *Science* **364**, 1255-1259, (2019).

18 Zhu, M. *et al.* Edge currents shunt the insulating bulk in gapped graphene. *Nat. Commun.* **8**, 14552, (2017).

19 Ying, J. *et al.* Magnitude and spatial distribution control of the supercurrent in Bi_2O_2Se-based Josephson junction. *Nano Lett.* **20**, 2569-2575, (2020).
